# Mention to Prof. Andrew V. Schally

**DOI:** 10.1007/s11154-025-09956-7

**Published:** 2025-03-17

**Authors:** Riccarda Granata

**Affiliations:** University of Turin, Via Giuseppe Verdi, 8, 10124 Torino, TO Italy

**Keywords:** Keywords, Andrew V. Schally, GHRH, GHRH agonists and antagonists, GHRH receptors

**Figure 1:**
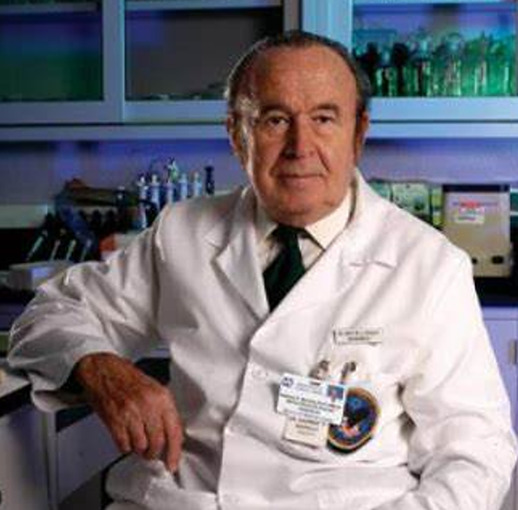
Andrew Viktor Schally Vilnius, 11/30/1926– Miami Beach 10/17/2024

This special issue is dedicated to Prof. Andrew Schally, who recently passed away at the age of 97. Prof. Schally and I had been invited by Prof. Casanueva, Editor-in-Chief of *REMD*, to serve as co-editors of this collection.

Prof. Schally was an innovator in the field of endocrinology and metabolism and one of the most influential scientists of the 20th century. He received the 1977 Nobel Prize in Physiology or Medicine for his discovery and characterization of hypothalamic neurohormones, which regulate hormone production and release by the pituitary gland, playing a vital role in controlling growth, reproduction, and other physiological functions. His research not only advanced our understanding of hormonal regulation but also paved the way for new treatments in reproductive health and cancer therapy.

Prof. Schally’s remarkable career includes over 2,200 publications in the most important peer-reviewed journals. In addition to receiving the Nobel Prize, he was honored with numerous prestigious awards, including the Albert Lasker Award, and the Golden Plate Award from the American Academy of Achievement.

For more than 30 years, Prof. Schally and his dedicated team of peptide chemists focused their research on the biological functions of growth hormone-releasing hormone (GHRH). Their extensive work led to the development of GHRH agonists and antagonists, which have shown therapeutic potential in a wide range of fields, including cardiovascular diseases, diabetes and metabolic disorders, oncology, neurodegenerative diseases, and regenerative medicine.

I had the honor and privilege of working with Prof. Schally—as I always called him, because “Doctor” felt too limiting, even though his collaborators used to call him “Dr. Schally”. I first reached out to him over ten years ago, after publishing several studies on the protective role of GHRH in the heart, to propose a collaboration on a new project on cardiac hypertrophy using his agonist, MR-409. At first, he was understandably skeptical, but after I sent him the results of our latest study, something ignited in him—his incredible scientific curiosity. That moment marked the beginning of a collaboration that grew stronger over the years, leading to publications not only in cardiology but also in oncology, with his peptide antagonists, and even in neurodegenerative diseases with MR-409. However, what struck me most was his intellectual honesty, his rigorous approach, his deep love for science, his unrelenting curiosity, and his kindness. If you earned his trust, he gave it fully, and that always amazed me, a man of his stature collaborating with a small research group like ours. When I sent Prof. Schally drafts of my manuscripts, he would print them out, meticulously correct them with his red pen, and send them back for revision—even fixing grammatical structures.

To me, Andrew Schally was like the father I never had the chance to know—himself a scientist—the guiding figure who gave me strength, enthusiasm, and the drive to always do more and better. He fueled my passion for science and my determination to understand and seek cures for the most challenging diseases. Over the years, we exchanged emails—which he called “letters”—where, beyond scientific discussions, we talked about sports, music, and good food. These conversations filled me with joy and warmed my heart. Recently, I had developed the habit of sending him and his dear wife, Lourdinha, packages containing pasta, tuna, olives, and other Italian specialties. Lourdinha later confessed to me that he particularly loved the tuna and continued eating it even as his health declined.

Like all his closest collaborators, I was deeply affected by his sudden, though not unexpected, passing. It was a loss beyond what I could have imagined. I lost not only the most significant mentor of my scientific career but also someone I deeply cared for, almost like a father.

For this reason, I dedicate each of my new projects to him, just as I dedicate this special collection of reviews on GHRH and its analogs in his honor.

When Prof. Felipe Casanueva, who was both a friend and collaborator of Andrew Schally long before me, proposed this collection, I accepted immediately with great enthusiasm. The hardest part of this work has been writing this dedication.

## Editorial

### GHRH: The first 50 years

Half a century has passed since the discovery of growth hormone-releasing hormone (GHRH), a groundbreaking discovery that revolutionized our understanding of endocrine regulation and growth hormone (GH) physiology. From its initial discovery in pancreatic tumors associated with acromegaly [1] to the cloning of its receptor in the early 1990s [2, 3], GHRH research has continually evolved.

Initially identified for its pivotal role in stimulating GH secretion from the anterior pituitary, GHRH has since been recognized as far more than a simple hypophysiotropic peptide. Over the decades, research has revealed its widespread influence beyond the neuroendocrine axis, with implications in metabolism, immune function, cardiovascular health, neuroprotection, and cancer biology [4, 5].

The identification of its diverse receptor splice variants and extrapituitary functions has expanded our knowledge of its biological significance. Today, we understand that GHRH and its receptor system operate beyond the pituitary, exerting autocrine and paracrine effects that regulate cell proliferation, apoptosis, inflammation, and tissue repair. These findings have paved the way for the development of synthetic GHRH agonists and antagonists, which hold great promise for therapeutic applications in conditions ranging from GH deficiency and metabolic disorders to cancer, cardiovascular and neurodegenerative diseases [5].

As we celebrate 50 years of GHRH research, this special issue of *REMD* offers a comprehensive overview on the past, present, and future of this remarkable neuropeptide. Bringing together leading experts, it explores key aspects of GHRH, including its molecular mechanisms, physiological functions, and translational potential. This issue presents contributions that examine the regulatory mechanisms governing GHRH function and GH secretion, along with the complex interactions between hypothalamic, pituitary, and peripheral signals and their associated signaling pathways [6–8]. Furthermore, it provides a comprehensive overview of the activation, regulation, molecular mechanisms, and signaling pathways of GHRH-Rs and their splice variants in various tissues [9]. The history and clinical applications of GHRH testing, along with challenges like obesity, hypothalamic damage, and aging, are also explored. Combination tests, such as GHRH with arginine) or GH secretagogues, improved accuracy and gained clinical acceptance [10].

The paper by Recinella et al. offers an in-depth analysis of GHRH deficiency and its broad impact on both central and peripheral tissues, highlighting its role in neurobehavioral functions, metabolism, immune regulation, and pain perception [11]. The use of genetically engineered mouse models has been crucial in uncovering the diverse functions of GHRH and elucidating its intricate relationship with GH.

The potential therapeutic applications of GHRH analogs are described in the review by Schally et al. [12]. Prof. Andrew Schally and his team of chemists have indeed developed both agonistic and antagonistic molecules of GHRH, which showed promise in regenerative medicine, aiding tissue repair, cardiac function, islet cell survival in diabetes and neuroprotection [5]. In fact, GHRH and its analogs, especially MR class agonists like MR-409, enhance myocardial function by improving contractility, mitigating oxidative stress and inflammation, and inhibiting pathological remodeling. Studies in animal models have demonstrated their efficacy in various cardiomyopathies, highlighting their therapeutic potential [13].

Importantly, GHRH and its analogs also promote the survival of insulin-producing pancreatic β-cells in both in vitro experiments and animal models. These beneficial effects highlight the potential of GHRH agonists and antagonists for clinical applications in treating human metabolic diseases or improving β-cell survival in transplantable cells [14]. Furthermore, GHRH agonist MR-409 exerts neuroprotective functions and enhances neurological recovery following ischemic stroke by activating extrapituitary GHRH-R signaling and stimulating endogenous NSC-derived neuronal regeneration. Additionally, MR-409 ameliorates the pathological features of spinal muscular atrophy (SMA) and muscle atrophy [15].

Meanwhile, GHRH antagonists exhibit anticancer and anti-inflammatory properties, inhibiting tumor growth and modulating immune responses [16, 17]. Preclinical studies suggest GHRH antagonists as a low-toxicity cancer therapy, particularly in lung, prostate, breast, gastrointestinal cancers as well as acute myeloid leukemia and brain tumors [15, 16, 18, 19].

GHRH and its analogs have significant impacts on the vascular system [20]. GHRH and its agonists stimulate bone marrow-derived stem cells to enhance angiogenesis, reduce vascular smooth muscle cells ossification, and ultimately inhibit vascular calcification. Additionally, GHRH agonists and antagonists influence endothelial cells and immune cells, such as macrophages, exerting anti-inflammatory and antioxidant effects that help maintain vascular endothelial integrity [17, 20].

Among the various reviews, Pérez-Gómez et al. examine the GHRH/GHRH-R hormone axis as a key regulator of gonadal function. The authors also explore the presence of GHRH and GHRH-R in reproductive systems across different species and their potential physiological roles. Additionally, they discuss how reproductive disorders, such as infertility, endometriosis, and hormone-related cancers like prostate and ovarian cancer, could benefit from hormonal interventions targeting the GHRH axis [21].

Overall, GHRH analogs are emerging as versatile therapeutic agents with significant implications across multiple fields.

We sincerely appreciate the contributions of all authors, reviewers, and researchers involved in this issue, as well as the broader scientific community for their ongoing efforts in advancing our understanding of GHRH. As we reflect on five decades of discovery, the story of GHRH is far from complete. With emerging research uncovering novel functions and potential clinical applications, the next 50 years promise even greater advancements in this field. We hope that this special issue not only honors the legacy of GHRH research but also inspires new investigations into its untapped potential.

## Data Availability

No datasets were generated or analysed during the current study.

## References

[1] Guillemin R, et al. Growth hormone-releasing factor from a human pancreatic tumor that caused acromegaly. Science. 1982;218:585–7. 6812220 10.1126/science.6812220

[2] Mayo KE. Molecular cloning and expression of a pituitary-specific receptor for growth hormone-releasing hormone. Mol Endocrinol. 1992;6:1734–44. 1333056 10.1210/mend.6.10.1333056

[3] Gaylinn BD, et al. Molecular cloning and expression of a human anterior pituitary receptor for growth hormone-releasing hormone. Mol Endocrinol. 1993;7:77–84. 7680413 10.1210/mend.7.1.7680413

[4] Schally AV, et al. Actions and potential therapeutic applications of growth hormone-Releasing hormone agonists. Endocrinology. 2019;160:1600–12. 31070727 10.1210/en.2019-00111

[5] Granata R, et al. Growth hormone-releasing hormone and its analogues in health and disease. Nat Rev Endocrinol. 2025;21:180–95. 10.1038/s41574-024-01052-1. 10.1038/s41574-024-01052-1 39537825

[6] Carlos D, Miguel L, Felipe C, Hypothalamic GHRH. Reviews Endocr Metabolic Disorders. 2025. 10.1007/s11154-025-09951-y. 10.1007/s11154-025-09951-y PMC12137398 39913072

[7] Montero-Hidalgo AJ, Rio-Moreno MD, Perez-Gomez JM, Luque RM, Kineman RD. Update on regulation of GHRH and its actions on GH secretion in health and disease. Rev Endocr Metab Disord. 2025. 10.1007/s11154-025-09943-y. 39838154 10.1007/s11154-025-09943-y

[8] Bioletto F, et al. Central and peripheral regulation of the GH/IGF-1 axis: GHRH and beyond. Rev Endocr Metab Disord. 2024. 10.1007/s11154-024-09933-6. 39579280 10.1007/s11154-024-09933-6

[9] Halmos G, Szabo Z, Dobos N, Juhasz E, Schally AV. Growth hormone-releasing hormone receptor (GHRH-R) and its signaling. Rev Endocr Metab Disord. 2025. 10.1007/s11154-025-09952-x. 39934495 10.1007/s11154-025-09952-x PMC12137518

[10] Caputo M, et al. The history of an effective, specific and sensitive diagnostic test: the GHRH test in clinical practice. Rev Endocr Metab Disord. 2024. 10.1007/s11154-024-09938-1. 39681762 10.1007/s11154-024-09938-1

[11] Recinella L, et al. Effects of growth hormone-releasing hormone deficiency in mice beyond growth. Rev Endocr Metab Disord. 2024. 10.1007/s11154-024-09936-3. 39695049 10.1007/s11154-024-09936-3

[12] Schally AV, Cai R, Zhang X, Sha W, Wangpaichitr M. The development of growth hormone-releasing hormone analogs: therapeutic advances in cancer, regenerative medicine, and metabolic disorders. Rev Endocr Metab Disord. 2024. 10.1007/s11154-024-09929-2. 39592529 10.1007/s11154-024-09929-2 PMC12137413

[13] Dulce RA, et al. Growth hormone-releasing hormone signaling and manifestations within the cardiovascular system. Rev Endocr Metab Disord. 2025. 10.1007/s11154-024-09939-0. 39883351 10.1007/s11154-024-09939-0 PMC12137388

[14] Steenblock C, Bornstein SR. GHRH in diabetes and metabolism. Rev Endocr Metab Disord. 2024. 10.1007/s11154-024-09930-9. 39560873 10.1007/s11154-024-09930-9 PMC12137473

[15] Liu Y, et al. GHRH and its analogues in central nervous system diseases. Rev Endocr Metab Disord. 2024. 10.1007/s11154-024-09920-x. 39470866 10.1007/s11154-024-09920-x

[16] Gesmundo I, et al. Growth hormone-releasing hormone and cancer. Rev Endocr Metab Disord. 2024. 10.1007/s11154-024-09919-4. 39422787 10.1007/s11154-024-09919-4

[17] Siejka A, Lawnicka H, Fakir S, Barabutis N. Growth hormone - releasing hormone in the immune system. Rev Endocr Metab Disord. 2024. 10.1007/s11154-024-09913-w. 39370499 10.1007/s11154-024-09913-w PMC11973240

[18] Munoz-Moreno L, Roman ID, Bajo AM. GHRH and the prostate. Rev Endocr Metab Disord. 2024. 10.1007/s11154-024-09922-9. 39505776 10.1007/s11154-024-09922-9

[19] Costoya J, Gaumond SI, Chale RS, Schally AV, Jimenez JJ. A novel approach for the treatment of AML, through GHRH antagonism: MIA-602. Rev Endocr Metab Disord. 2024. 10.1007/s11154-024-09917-6. 39417961 10.1007/s11154-024-09917-6 PMC12137468

[20] Yu H, Peng H. Effects of GHRH and its analogues on the vascular system. Rev Endocr Metab Disord. 2024. 10.1007/s11154-024-09932-7. 39570567 10.1007/s11154-024-09932-7

[21] Perez-Gomez JM, Montero-Hidalgo AJ, Luque RM. GHRH and reproductive systems: mechanisms, functions, and clinical implications. Rev Endocr Metab Disord. 2024. 10.1007/s11154-024-09931-8. 39612161 10.1007/s11154-024-09931-8

